# Engineering of the fast-growing cyanobacterium *Synechococcus* sp. PCC 11901 to synthesize astaxanthin

**DOI:** 10.1186/s13068-025-02626-5

**Published:** 2025-02-28

**Authors:** Nico Betterle, Eliana Gasparotto, Elia Battagini, Edoardo Ceschi, Francesco Bellamoli, Peter J. Nixon, Matteo Ballottari

**Affiliations:** 1 Department of Biotechnology, University of Verona, Strada Le Grazie 15, 37134 Verona, Italy; 2 Department of Life Sciences, Sir Ernst Chain Building-Wolfson Laboratories, Imperial College London, South Kensington Campus, London, SW7 2AZ UK

**Keywords:** Metabolic engineering, Antioxidants, Ketocarotenoids, Microalgae, Cyanobacteria, Photosynthesis, CO2 consumption

## Abstract

**Background:**

Astaxanthin is a red pigment required by feed, nutraceutical, and cosmetic industries for its pigmentation and antioxidant properties. This carotenoid is one of the main high-value products that can nowadays be derived from microalgae cultivation, raising important industrial interest. However, state-of-the-art astaxanthin production is the cultivation of the green alga *Haematococcus pluvialis* (or *lacustris*), which faces high costs and low production yield. Hence, alternative and efficient sources for astaxanthin need to be developed, and novel biotechnological solutions must be found. The recently discovered cyanobacterium, *Synechococcus* sp. PCC 11901 is a promising photosynthetic platform for the large-scale production of high-value products, but its potential has yet to be thoroughly tested.

**Results:**

In this study, the cyanobacterium *Synechococcus* sp. PCC 11901 was engineered for the first time to our knowledge to produce astaxanthin, a high-value ketocarotenoid, by expressing recombinant β-ketolase (bKT) and a β-hydroxylase enzymes (CtrZ). During photoautotrophic growth, the bKT-CtrZ transformed strain (called BC) accumulated astaxanthin to above 80% of the total carotenoid. Moreover, BC cells grew faster than wild-type (WT) cells in high light and continuous bubbling with CO_2_-enriched air. The engineered strain reached stationary phase after only 4 days of growth in an airlift 80-mL photobioreactor, producing 7 g/L of dry biomass, and accumulated ~ 10 mg/L/day of astaxanthin, which is more than other CO_2_-consuming multi-engineered systems. In addition, BC cells were cultivated in a 330-L photobioreactor to link lab-scale experiments to the industrial scale-up.

**Conclusions:**

The astaxanthin volumetric productivity achieved, 10 mg/L/day, exceeds that previously reported for *Haematococcus pluvialis,* the standard microalgal species nowadays used at the industrial level for astaxanthin production, or for other microalgal strains engineered to produce ketocarotenoids. Overall, this work identifies a new route to produce astaxanthin on an industrial scale.

**Supplementary Information:**

The online version contains supplementary material available at 10.1186/s13068-025-02626-5.

## Background

Due to their ability to perform oxygenic photosynthesis, eukaryotic microalgae and prokaryotic cyanobacteria are attractive carbon-neutral biological platforms for the industrial production of bioproducts using sunlight, water, and CO_2_. However, two main drawbacks limit the industrial exploitation of microalgae: low cell densities and moderate growth rates [1]. These two factors are often critical for the economic sustainability of algae production, which is already burdened by high maintenance and processing costs. Therefore, finding suitable strains for large-scale industrial use is of utmost importance.

The recently isolated cyanobacterium *Synechococcus* sp. PCC 11901 (hereafter *Syn11901*) possesses suitable properties for large-scale cultivation [2]. *Syn11901* has a fast growth rate, grows to high cell densities and biomass yields, tolerates high salinity, and has a range of synthetic biology tools available for genetic engineering [2–5].

One strategy to offset the high processing costs of growing microalgae and cyanobacteria at the industrial scale is to produce high-value products. In particular, natural products found in microalgae are an attractive target [6], especially antioxidants, which can be used in nutraceutical, cosmetic, and feed formulations [7].

Among the natural antioxidants, astaxanthin (hereafter referred to as Asta) is a valuable molecule for relevant industrial applications [8] with its price reaching 60,000 €/kg. The main routes to obtain natural Asta are from bacteria such as *Paracoccus carotinifaciens* [9] or from photoautotrophic microalgae [10] such as *Haematococcus pluvialis*, also named *Haematococcus lacustris* [10, 11] and, to a lesser extent, *Chromochloris zofingiensis* [12].

The use of *H. pluvialis* does, however, have some limitations, notably the requirement of two different growth conditions for either growing green biomass or accumulating Asta, the latter being induced by stress conditions such as high light, high/low temperature, and/or nutrient starvation [13]. Moreover, Asta accumulated in *H. pluvialis* has a low bio-accessibility due to the thick cell wall, making mechanical disruption necessary to release Asta for human or animal consumption [14], a process that increases production costs.

Advances in synthetic biology have led to the generation of genetically modified cyanobacteria and microalgae that synthesize Asta and, importantly, overcome some of the limitations of *H. pluvialis* strains [15–21]. The production of Asta in cyanobacteria [15–18, 20, 21] is also facilitated by the abundant accumulation of β-carotene and zeaxanthin (hereafter βcar and Zea, respectively), both precursors of the Asta pathway [8, 22]. Cyanobacteria have the additional advantage that carotenoids are more easily extracted from biomass than *H. pluvialis* [22].

However, the use of cyanobacteria to produce Asta has until now been undermined by the low accumulation of biomass in lab-scale experiments [15, 21]. For all these reasons, the feasibility of producing Asta in *Syn11901* was herein investigated for the first time to our knowledge. The results obtained demonstrate a novel biotechnological solution for Asta production in unicellular photosynthetic organisms: engineered *Syn11901* can produce Asta at unprecedented levels in cyanobacteria, highlighting its potential route for the industrial production of Asta.

## Material and methods

### Cyanobacterial strains and cultivation

The cyanobacterium *Syn11901* [2] was used as the experimental strain in this work and is referred to as the wild type (WT). Cells were maintained on AD7 solid agar medium containing 10 mM glycerol [2], whereas liquid cultures were grown in flasks using the Modified AD7 medium (hereafter named MAD medium), as previously described [2]. MAD medium used for flask cultivation was supplemented with 10 mM NaHCO_3_ as a C source. High-density cultivations were conducted in batch mode in 80 mL airlift photobioreactors (PBRs) in the Multicultivator system MC-1000-OD (PSI, Czech Republic) with independent white light-emitting diodes (LEDs) illumination and temperature control at 35 °C. This device automatically monitors the optical density at 720 nm as an index of cell concentration. The optical density of the cell cultures was, in addition, measured at 720 nm upon sampling and diluting the samples to avoid the saturation of OD measurement. Before starting the growth tests, cyanobacterial cells with OD at 720 nm (OD_720_) of 0.3 were acclimated for 2 days in the PBRs with illumination of 250 micromoles of photons per square meter per second (µmol/m^2^/s) and a constant supply of CO_2_ (3% CO_2_-enriched air). Each culture was then diluted into fresh MAD medium to an OD_720_ of 0.1 before starting the growth experiments. Growth started with low light conditions (250 μmol photons/m^2^/s) for cell acclimation, then (after 12 h) shifted to higher light conditions (up to 2250 μmol/m^2^/s) as described in the text with a constant supply of CO_2_ (3% CO_2_ mixed with air). Growth in nutrient starvation was induced by growing cells in MAD medium prepared with the absence of a specific nutrient such as nitrate (MAD-N), phosphate (MAD-P) or iron (MAD-Fe). In particular, cells grown as described above at 2250 μmol/m^2^/s of light were harvested at stationary phase by centrifugation, washed once with the growth medium adopted for the following cultivation steps, and diluted to OD_720_ 3 in MAD-N, MAD-P, MAD-Fe or MAD as control. Cells in the different growth media were then cultivated in batch in 80-ml airlift PBRs in the multicultivator system with illumination of 2250 µmol/m^2^/s and a constant supply of CO_2_ (3% CO_2_-enriched air). Large-scale growth was performed in batch mode using a 330-L PBR (Technology Farm, Italy) of diameter 50 cm, with light provided by 4 LED white light strips contained in a plastic cylinder positioned inside the PBR (~ 800 µmol/m^2^/s measured at the internal surface) and by six external red/white strips (~ 550 µmol/m^2^/s measured at the outer surface) placed outside the PBR. After 3 days of cyanobacterial cultivation, an external LED panel was placed close to the right side of the PBR to increase the light supply. Before cultivation, cells were transferred from 25-ml flasks in exponential phase in a 4-L bottle containing MAD medium obtaining as initial OD_720_ of 0.02. The cells were kept at 37 °C, illuminated with a white LED lamp (~ 200 µmol/m^2^/s), and air-lifted with a pump. Once the culture reached an OD_720_ of 2.5, it was used as an inoculum for the industrial PBR. The photobioreactor was filled with purified and UV-treated water, whereas nutrient salts were manually added inside the PBR. The final composition was the same as the MAD medium but included two modifications for economic reasons: a tenfold reduction in tris(hydroxymethyl)aminomethane (Tris) buffer concentration (1.03 mM, pH 8.2) and the use of one-third of vitamin B12 (~ 1.3 ng/L). In addition, 10 mM NaHCO_3_ was added to the medium. The temperature was kept between 33 and 35 °C, and the pH was maintained between 8.2 and 8.5. Air-bubbling was supplied to mix the cells and provide some CO_2_. Every hour, ~ 10 s of pure CO2 was automatically provided to restore the pH to 8.2. Biomass accumulation was followed by daily measurements of OD_720_.

### Generation of recombinant constructs and cyanobacterial transformation

The gene sequences encoding the β-carotene 4-ketolase of *Chlamydomonas reinhardtii* (AY860820.1, *bKT*) and the β-carotene hydroxylase of *Brevundimonas* sp. SD212 (MK214313.1, *crtZ*) were taken from the literature [17, 19]. The sequences were codon-optimized for expression in *Syn11901* using an open software system (https://www.idtdna.com/CodonOpt) with the codon usage of *Synechococcus* sp. PCC 7002, a close model species, as a reference [2]. Synthetic sequences were generated by Eurofins Genomics (Ebersberg, Germany). Sequences of *smR* and *kmR* selection cassettes, conferring resistance to spectinomycin and kanamycin, respectively, were amplified from pSW039 and pSW071 plasmids [2] in the order given. Such plasmids were obtained from the Addgene repository (https://www.addgene.org). Similarly, the flanking sequences required for homologous recombination in the *acsA* locus were taken from plasmid pSW039. *P*_*cpt*_ promoter and T7 terminator sequences were amplified from plasmid pSW036 and pSW071, respectively. The DNA sequences used for metabolic engineering can be found at this link: 10.5281/zenodo.13234572. DNA constructs were generated using Gibson Assembly by Thermo Fisher (Waltham, USA), followed by the transformation of *Escherichia coli* TOP10 chemically competent cells.

*Syn11901* transformations were carried out using established protocols for transforming *Synechocystis* sp. PCC 6803 [23, 24]. A *Syn11901* culture in the exponential phase was first resuspended in fresh MAD medium supplemented with 10 mM NaHCO_3_ without antibiotic selection to a final OD_720_ of 2.5. Then, a 1.5-mL aliquot was transferred to a 12-well microtiter plate, and 1 µg of the desired linearized plasmid DNA was added. The plate was kept overnight in a 37 °C chamber with stirring to avoid cell precipitation and illuminated using a white light LED panel (~ 100 µmol photons/m^2^/s). 16 h later, cells were plated on AD7 solid medium containing the required antibiotic for selection, as indicated in previous works [2, 3].

### Genomic DNA PCR analysis of Syn11901 transformants

Genomic DNA templates were prepared as previously described [23]. Cells resuspended in 20 μL aliquot of Milli-Q water were mixed with an equal volume of 100% ethanol, followed by brief vortexing. A 200-μL aliquot of a 10% (w/v) Chelex-100 Resin (BioRad, USA) suspension in water was added to the sample before mixing and heating at 95 °C for 10 min to lyse the cells. Following centrifugation at 16,000 g for 10 min to pellet the cell debris, 5 μL of the supernatant was used as a genomic DNA template in a 25 μL PCR reaction mixture. Phusion DNA polymerase (Thermo Fisher, USA) was used for genomic DNA PCR analyses. A list of primers is given in Table 1. Transgenic DNA copy homoplasmy in *Syn11901* was tested using suitable primers listed in the Supplemental Materials. The genomic DNA location of these primers is indicated in Figs. 2 and 3 for the appropriate DNA constructs. A similar approach was conducted to monitor the stability of the genetic modifications introduced, revealing stable genotypes of *Syn11901* transformants for up to two years.

**Table 1:** Sequence of oligonucleotide primers used in the present work

Oligos name	Oligos DNA sequence
*acsA-5' fw*	ctcctagggttgggtttga
*acsA-3' rv*	cgcagattggtgcatttat
*acsA rv*	tgtcccatttctcaaaccatt
*bKT rv*	tgcgtttatcagaatcttcc
*bKT fw*	tgttcattaccacgcacgac
*acsA-fl3' rv*	gcctttatcgaagggaacta
*rnpA-RT fw*	ggacttgccgtctacctcac
*rnpA-RT rv*	ctttgcggctgatgctgatg
*bKT-RT fw*	tttatgactgcggcaccgat
*bKT-RT rv*	atctgccgacactttgggag
*crtZ-RT fw*	tcttgacagcgtttctcggg
*crtZ-RT rv*	tgctgcgaacactactgcaa

### RNA extraction and reverse transcription

RNA extraction was executed using TRIzol reagent (Thermo Fisher, USA), following instructions provided by the manufacturer. Each extraction was carried out starting from a cell pellet derived from 7 ml of a cyanobacterial culture in exponential phase brought to a final OD_720_ of 3.5. The RNA concentration was determined using a NanoDrop spectrophotometer (Thermo Fisher, USA). Total RNA was reverse transcripted to cDNA using RevertAid reverse transcriptase (Thermo Fisher, USA) following the manufacturer's instructions. PCR analysis using cDNA was performed using Phusion DNA polymerase (Thermo Fisher, USA), with 30 cycles of amplification.

### Protein analysis

Cells in the mid-exponential growth phase (OD_720_ ~ 1) were harvested by centrifugation at 4,000 g for 10 min. The pellet was resuspended in a solution buffered with 25 mM Tris–HCl, pH 8.2, containing 1 mM benzamidine, 5 mM ε-aminocaproic acid, and 1 mM phenylmethylsulfonyl fluoride (PMSF) as protease inhibitors. Cells were lysed with a Cell Disruptor (Constant System Limited, UK), set at 37,500 kpsi. A slow-speed centrifugation (350x*g* for 5 min) was applied to remove unbroken cells. For protein electrophoretic analysis, sample extracts were solubilized upon incubation for 1 h at room temperature in the presence of 125 mM Tris–HCl, pH 6.8, 3.5% (w/v) sodium dodecyl sulfate (SDS), 10% (w/v) glycerol, 2 M urea, and 5% β-mercaptoethanol. Sodium dodecyl sulfate–polyacrylamide gel electrophoresis (SDS-PAGE) was performed using a Mini-PROTEAN gel system (BIORAD, USA). Western blot analysis entailed the transfer of separated proteins to a 0.45-μm pore size PVDF membrane (Life Technologies, USA), followed by immunoblotting with rabbit anti-His-tag specific polyclonal antibodies (Sigma-Aldrich, USA) and detection with chemiluminescent western blotting kit (Sigma-Aldrich, USA).

### Pigment extraction and absorption spectra and HPLC analyses

Cyanobacterial cultures were centrifuged at 10,000 × g for 5 min, and pigments were extracted using dimethyl sulfoxide (DMSO). An incubation time of at least 90 min in a rotating mixer facilitated complete pigment extraction. Extracts were then diluted in acetone 95%, with the latter previously buffered with Na_2_CO_3_, to a final acetone concentration of 80%. The absorption spectra of the pigment extracts were measured with the Jasco V-730 spectrophotometer in the visible range (350–750 nm). Then, DMSO-acetone spectra were fitted with the different pigment absorption spectral forms [25]. Reversed-phase HPLC analysis was conducted as previously described [19].

### Thin-layer chromatography analysis

Pigments were extracted in isopropanol from a cell pellet of transformed cyanobacteria, and then they were separated by thin-layer chromatography (TLC) on Silica Gel 60 F254 Coated Aluminum-Backed TLC Sheets (Sigma-Aldrich, USA) using an appropriate mobile phase (60% hexane, 20% chloroform, 20% acetone). The TLC fraction predicted as Asta was collected and eluted in isopropanol. Upon elution, the sample was dried in a speed vacuum system.

### Mass spectroscopy analysis

The dried sample obtained by TLC was resuspended in methanol and loaded on HPLC in tandem with an Orbitrap mass spectrometer. Data acquisition was performed in full scan mode in the mass range of 50–800 m/z. The full description of the MS results can be found at this link: 10.5281/zenodo.14283202.

### Dry weight measurement

For dry biomass weight measurement, 30 mL of culture from each airlift PBR in the multicultivator system was collected into pre-weighed tubes. For the 330-L PBR, 50 mL of culture was taken for the dry weight analysis. Cells were then pelleted by centrifugation (4500 g, 15 min), and the supernatants were discarded. Pellets were washed once with milliQ water before transferring the tubes to a lyophilization system for freeze drying under vacuum. Samples were weighed again to obtain the dry weight.

### Statistical analysis

The statistical significance was evaluated by comparing results obtained in the same experiment running Tukey–Kramer multiple comparison tests. Statistically significant variations with a *p* < 0.05 are marked with different letters.

## Results

### Heterologous expression of beta-carotene 4-ketolase in *Synechococcus sp.* PCC 11901

**Fig. 1:**
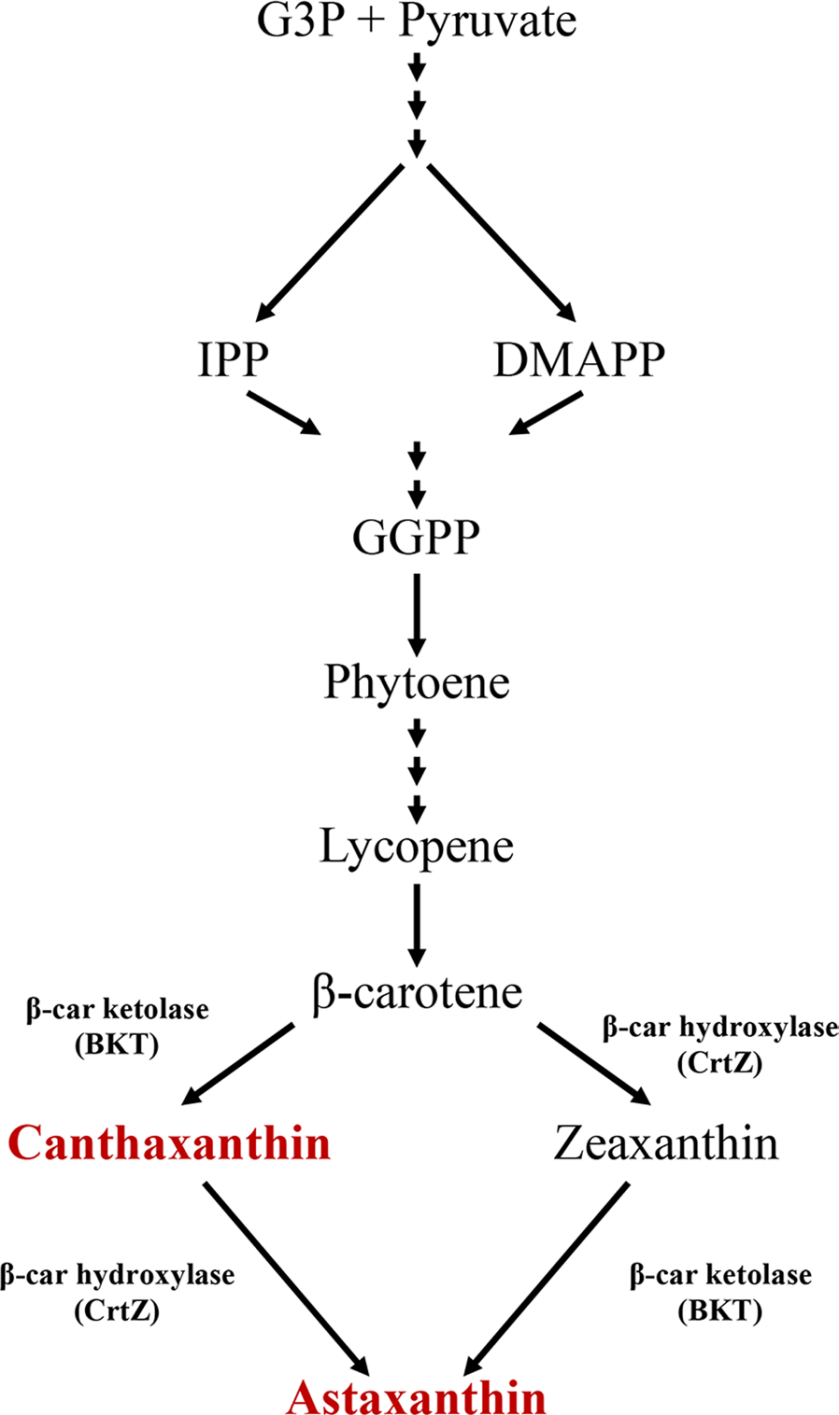
Schematic view of astaxanthin biosynthesis. Ketocarotenoids are reported in red. The enzymes required for conversion of βcar into ketocarotenoids are reported. *G3P* glyceraldehyde 3-phosphate, *IPP* isopentenyl diphosphate, *DMPP* dimethylallyl diphosphate

Previous work has shown that the synthesis of Asta in cyanobacteria can be achieved by heterologous expression of prokaryotic ketolase and hydroxylase enzymes, as reported in Fig. 1 [15, 17, 18, 21]. In the case of the green alga, *Chlamydomonas reinhardtii*, the constitutive overexpression of just the endogenous β-carotene 4-ketolase gene (Uniprot Q4VKB4), hereafter referred to as Cr-bKT was sufficient to convert both βcar and Zea to Asta (Fig. 1), achieving high accumulation of the latter [19].

**Fig. 2:**
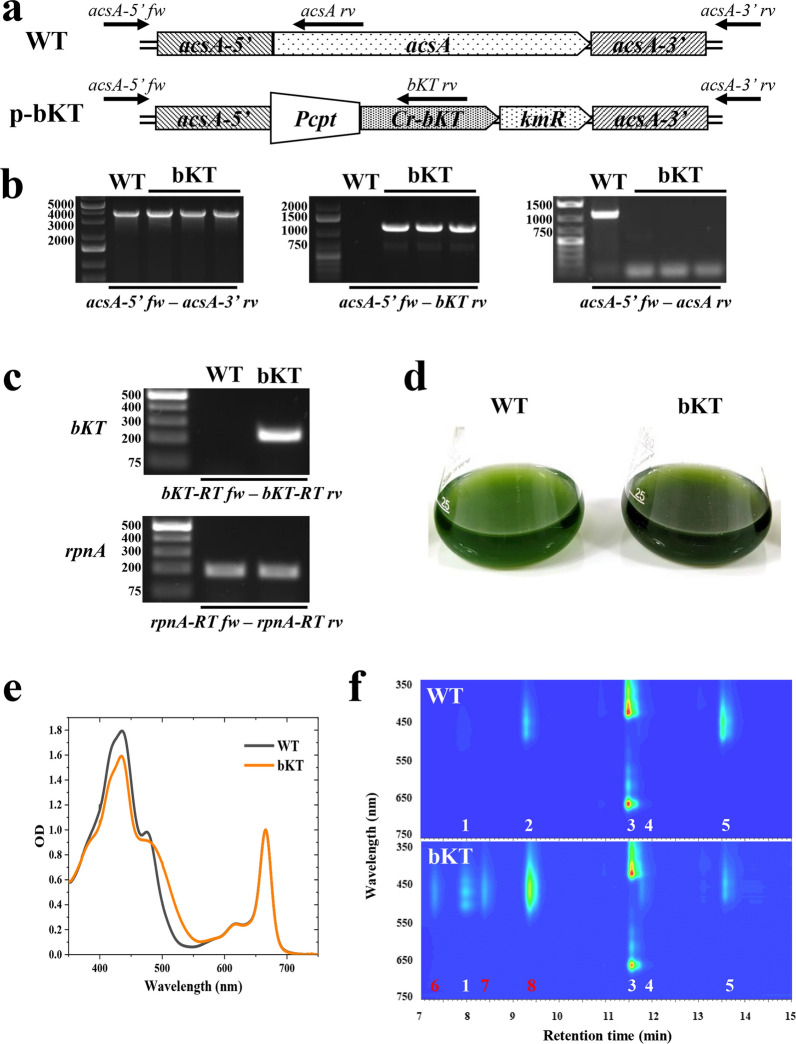
Generation of the bKT transformant and pigment analysis. **a** Schematic of the p-bKT DNA construct, harboring flanking sequences for the replacement of the *acsA* locus by homologous recombination [2]. The two heterologous genes, namely the *bKT* gene from *Chlamydomonas reinhardtii* [19] and the kanamycin-resistance cassette (*kmR*), were in an operon configuration and under the control of the *P*_*cpt*_ constitutive promoter. **b** Genomic DNA PCR analysis using primers *acsA-5’ fw* and *acsA-3’ rv* (left panel). The expected size of the PCR products was 3767 and 3820 bp in WT and bKT lines, respectively. Genomic DNA PCR analysis using primers *acsA-5’ fw* and *bKT rv* (middle panel). The expected size of the PCR product in bKT samples was 1168 bp, whereas no amplification was expected in the WT sample. A faint byproduct of 750 bp was also present in the bKT samples. Genomic DNA PCR analysis using primers *acsA-5’ fw* and *acsA rv* (right panel). The expected size of the PCR product in the WT sample was 1107 bp, whereas no amplification should occur in the bKT sample. **c** RT-PCR analysis for the verification of the expression of transgenic *bKT* gene and constitutive *rpnA* gene transcripts in WT and bKT cell cultures. The PCR products were separated on 1.5% agarose gel. The expected size of the PCR products was 187 and 231 bp for *rpnA* and *bKT* gene transcripts, respectively. **d** Pigmentation of WT and bKT photoautotrophic cultures grown in flasks. **e** Absorption spectra in the visible light range (350–750 nm) of pigment extracts from WT and bKT cultures shown in **C**. **f** HPLC analyses of microalgal pigments from WT and bKT cultures shown in **d**. 1, Mixoxanthophyll; 2, zeaxanthin; 3, chlorophyll *a*; 4, echinenone; 5, β-carotene; 6, 3S,3’S trans-astaxanthin; 7, 3S,3’S 9-cis-astaxanthin; 8, canthaxanthin

To evaluate the ability of the Cr-bKT enzyme alone to drive the synthesis of Asta in *Syn11901* as in the case of *Chlamydomonas reinhardtii*, a DNA construct was designed for the transformation of wild-type *Syn11901* (WT) through double homologous DNA recombination at the *acsA* locus, identified previously as a suitable site for insertion of foreign DNA [2]. Construct p-bKT (Fig. 2a) was codon-optimized for expression in *Syn11901* and designed to replace the *acsA* gene. To achieve this, the sequence encoding the native chloroplast transit peptide sequence was deleted from *Cr-bKT,* and a kanamycin-resistance cassette (*kmR*) was inserted downstream in the same operon. Expression of the two heterologous genes was controlled by the constitutive *P*_*cpt*_ promoter [2, 26]. The nucleotide sequence of this construct is described in the DNA constructs section of Supplementary Information.

PCR analysis confirmed the correct genomic insertion of the exogenous DNA construct into the acsA locus [2, 27] of the antibiotic-resistant strains. Primers *acsA-5’ fw* and *acsA-3’ rv* (Table 1) were designed according to the flanking regions of the selected locus (Fig. 2a). PCR amplification using WT genomic DNA as a template generated a product of 3,767 bp, while it generated a 3,820 bp amplicon in the bKT lines. Due to the similar sizes of the PCR products from WT and transformant lines, it was impossible to distinguish them clearly on agarose gel (Fig. 2b, left). Thus, the recombinant DNA sequence insertion in the bKT transformants genome was verified using primers *acsA-5’ fw* and *bKT-rv*, with the latter annealing specifically to the recombinant *bKT* gene. The expected size of the PCR product was 1168 bp (Fig. 2b, middle). No PCR product was obtained using WT DNA as a template, whereas bKT samples generated a product of the expected size. PCR amplification using primers *acsA-5’ fw* and *acsA rv* was conducted to verify the absence of WT sequences in the bKT samples. The predicted WT sequence of 1107 bp was amplified in the WT sample. In contrast, it was absent in the bKT samples (Fig. 2b, right), suggesting complete segregation of the transgenes in the transformants. A reverse transcriptase PCR (RT-PCR) analysis was performed to confirm the expression of the *bKT* gene transcript. Specific primers (Table 1) were designed to amplify sequences from *bKT* and *rpnA* gene transcripts: the latter was used as a reference, according to previous literature [28]. The predicted 187-bp DNA product from *rpnA* transcript was amplified in both WT and bKT samples. Conversely, only bKT strain expressed the mRNA encoding the bKT gene product (Fig. 2c), as evidenced by the amplification of the predicted DNA sequence of 231 bp.

WT and bKT cultures were grown photoautotrophically in shake flasks at an irradiance of ~ 100 µmol/m^2^/s of white light at 37 °C. Liquid MAD medium, specially formulated for growing *Syn11901* cultures [2], was supplemented with 100 mM NaHCO_3_ as a C source. A characteristic feature of WT and bKT cultures was their different pigmentation upon reaching the stationary phase (Fig. 2d): WT was green, whereas bKT had a darker/brownish hue. Absorption spectra of chlorophyll and carotenoids extracted from cells revealed a shoulder between 500 and 550 nm in bKT that was absent in WT samples (Fig. 2e), consistent with the accumulation of ketocarotenoids [15, 18, 19].

To confirm the presence of ketocarotenoids, HPLC analyses comparing WT and bKT extracts were conducted (Fig. 2f). The attribution of the pigments was based on evaluating the absorption spectra of eluted fractions obtained by reversed-phase HPLC (Supplementary Figure S1, Additional File 1), integrated with previous analyses conducted in the lab with engineered microalgal strains [19]. The WT extract comprised three primary pigments: chlorophyll *a* (Chl*a*)*,* Zea, and βcar. Traces of mixoxanthophyll (Mixo), a carotenoid glycoside possibly involved in cell-membrane structure and thylakoid organization in cyanobacteria [29], were also observed at ~ 8 min. By contrast, bKT samples showed a very different profile. No Zea was observed; instead, a large amount of canthaxanthin (Cantha) was present, resulting from the conversion of βcar by the heterologous bKT catalytic activity. Consequently, the βcar/Chl*a* ratio was lower in the bKT line compared to WT (Fig. 2f). 3S,3’S trans-astaxanthin (Asta), Mixo, and 3S,3’S 9-cis-astaxanthin (cisAsta) were also synthesized, although in minor amounts compared to Cantha.

Previous literature showed that levels of Zea increased in cells grown under high irradiance compared to cells grown under low irradiance [30, 31]. Since Zea is the substrate of bKT for the synthesis of Asta [8, 22], the level of Asta was investigated in bKT cells grown at high light intensity. To this aim, bKT cultures were grown in airlift 80-mL photobioreactors (PBRs) exposed to a higher light intensity than flask cultures described above, 1500 µmol/m^2^/s, and a 3% CO_2_-enriched air supply. Samples were taken at day 3 and day 7 (Supplementary Figure S2, Additional File 1), followed by pigment extraction and analysis by HPLC. Compared to the previous growth condition in shake flasks, bKT strains accumulated similar amounts of Asta (Supplementary Figure S2, Additional File 1), confirming that this was an inherent feature of the transformant since Asta content did not considerably change in cultures grown under high irradiance, where the metabolic flux toward terpenoids was possibly enhanced. Conversely, Cantha abundantly accumulated, indicating that bKT was acting prevalently on βcar and poorly on Zea.

Overall, these data suggested that the expression of the heterologous bKT from *C. reinhardtii* alone in *Syn11901* was insufficient to promote substantial remodeling of the terpenoid pathway toward the synthesis of Asta. The reason is probably due to differences in the activities of the endogenous hydroxylases: in *C. reinhardtii* this is sufficient to convert Cantha to Asta, whereas in cyanobacteria the hydroxylase activity is insufficient. It is worth noting that even in *C. reinhardtii* overexpression of a β-carotene hydroxylase enzyme strongly increased the Asta yield at the expense of Cantha [32].

### Heterologous expression of bKT and CrtZ in* Synechococcus sp.* PCC 11901

**Fig. 3:**
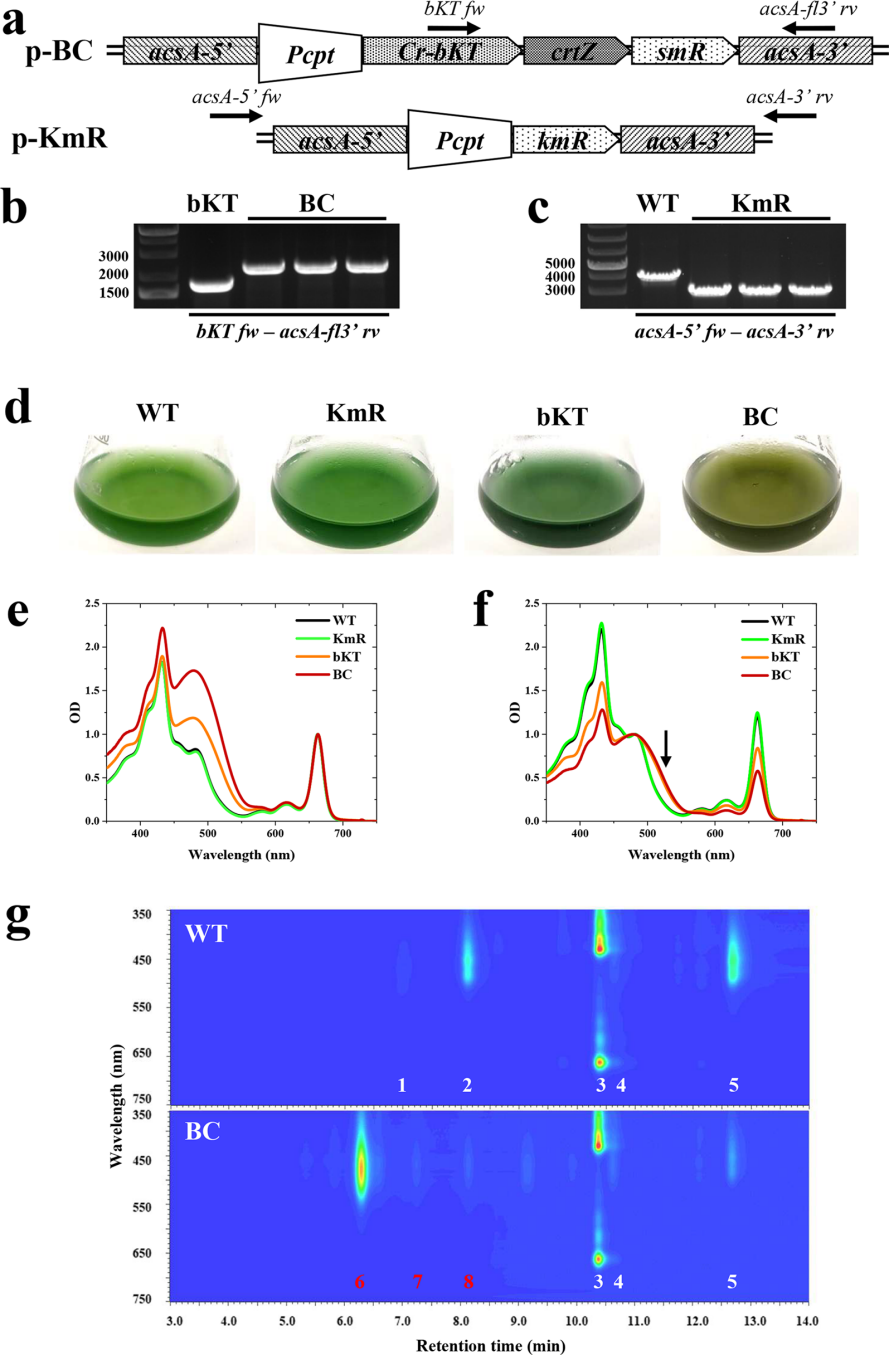
Generation of BC and KmR transformants and pigment analysis. **a** Schematic of p-BC and p-KmR DNA constructs. The first was designed for the replacement of the *kmR* gene in the bKT recipient strain by homologous recombination. The recombinant *crtZ* gene and the spectinomycin-resistance cassette (*smR*) were in an operon configuration, with the *bKT* gene as the leader gene, and under the control of the *P*_*cpt*_ constitutive promoter. The p-kmR construct was designed for replacing the *acsA* locus with the *kmR* gene only. **b** Genomic DNA PCR analysis of BC transformants and bKT recipient strain using primers *bKT fw* and *acsA-fl3’ rv*. The expected size of the PCR products was 1677 and 2181 bp in bKT and BC lines, respectively. **c** Genomic DNA PCR analysis of KmR lines and WT recipient strain using primers *acsA-5’ fw* and *acsA-3’ rv*. The expected sizes of the PCR products were 3767 and 2862 bp in WT and KmR lines, respectively. **d** Comparison of the pigmentation of WT, KmR, bKT, and BC cultures photoautotrophically grown in flasks. BC culture showed a brownish coloration, different from the blue-green WT and KmR cultures. **e** Absorption spectra in the visible range of pigment extracts from WT, KmR, bKT and BC lines. Spectra were normalized to the absorbance of chlorophyll *a*. **f** Absorption spectra in the visible range of pigment extracts from WT, KmR, bKT and BC lines. Spectra were normalized to the absorbance of carotenoids. Arrow indicates the shift of absorption attributed to the accumulation of Asta in the BC line. **g** Representative HPLC profiles of WT, bKT, and BC pigment extracts. 1, Mixoxanthophyll; 2, zeaxanthin; 3, chlorophyll *a*; 4, echinenone; 5, βcarotene; 6, 3S,3’S trans-astaxanthin; 7, mixoxanthophyll; 8, 3S,3’S 9-cis-astaxanthin

Previous work has shown that heterologous expression of *crtZ* gene from *Brevundimonas* sp. SD-212 in *Synechocystis* sp. PCC 6803 successfully converts Cantha to Asta [17, 33]. Based on this, the *crtZ* gene was codon-optimized for expression in *Syn11901* (see Materials and methods section) and inserted into the genome of *Syn11901* using a second DNA construct, named p-BC, which was designed to enhance Asta yield in *Syn11901* by co-expressing the bKT and CrtZ recombinant enzymes. In more detail, the p-BC construct harbored the same genetic elements present in the p-bKT construct but the *kmR* cassette was replaced by *crtZ* and a spectinomycin-resistance cassette (*smR*) in a single operon (Fig. 3a). In parallel, another construct was designed, harboring only the *kmR* cassette as a recombinant gene. The use of this last construct, referred to as p-KmR, was intended as a control for the replacement of *acsA* locus in the *Syn11901* genome.

Upon cyanobacterial transformation and growth in selective media, the genotype of the transformants was tested by PCR analysis (Fig. 3b, c). Genomic DNAs from three BC independent cultures were analyzed using *bKT fw* and *acs-fl3’ rv* primers. DNA from the bKT culture, the recipient strain of the p-BC construct, was used as a control. The latter strain generated a PCR product of 1677 bp, whereas the BC genomic DNAs generated a single PCR product of 2181 bp, confirming the correct replacement of the p-bKT construct with the p-BC construct (Fig. 3b). Similarly, PCR analyses were conducted to evaluate KmR transformants generated PCR products of 3767 bp and 2862 bp in WT control and KmR lines, respectively (Fig. 3c). This result confirmed complete segregation of the transgenes in the KmR transformants.

WT, KmR, bKT and BC photoautotrophic cultures were grown in shake flasks using ~ 100 µmol/m^2^/s of white light at 37 °C. The first two evidenced a similar green coloration, whereas BC liquid culture showed a brownish hue, more evident than the bKT strain (Fig. 3d), suggesting a different pigment composition.

### Astaxanthin productivity by photoautotrophic cultivation of engineered *Synechococcus sp.* PCC 11901

Samples from WT, KmR, bKT, and BC cultures were collected during exponential phase for pigment analysis. Absorption spectra in the visible region of pigment extracts from WT and the KmR control did not show major differences (Fig. 3e), as expected given the similar coloration of the liquid cultures (Fig. 3d). The spectrum from the BC strain showed a clear distinctive shoulder peaking at ~ 480 nm, in agreement with the accumulation of ketocarotenoids (Fig. 2e). This shoulder was even higher than the one observed in the bKT spectrum, possibly related to a greater value for the ratio of carotenoids to chlorophyll. Of interest was the comparison of the spectra normalized to the carotenoid content (Fig. 3f), which indicated that the absorption shoulder attributed to carotenoids was slightly shifted towards longer wavelengths in the BC extract compared to the case of bKT. This was corroborated by the evidence that, after pigment separation by HPLC, the maximum absorption peak of Cantha was 474 nm, whereas the maximum of Asta was red-shifted to 478 nm of (Supplementary Figure S1, Additional File 1).

Consistent with the absorption spectra, HPLC analyses confirmed that 3S,3’S trans-astaxanthin was the most abundant carotenoid in the BC line (Fig. 3g) and represented more than 70% of total carotenoid. No Zea was detected and only traces of Cantha were identified. βcar was poorly accumulated in both bKT and BC samples compared to WT. Traces of 3S,3’S 9-cis-astaxanthin were observed in the BC extract, probably as a side product of the Asta biosynthesis pathway.

To assess protein expression, total cell extracts from *Syn11901* WT and transformants were analyzed by SDS-PAGE followed by Coomassie blue staining or Western blot (Supplementary Figure S3a and b, Additional File 1). The most abundant polypeptides observed for all extracts were the α and β subunits of phycocyanin (~ 15 kDa), in accordance with other cyanobacteria [34, 35]. Heterologous bKT, CrtZ, KmR, and SmR have predicted molecular masses of ~ 31, ~ 17, ~ 28 and ~ 26 kDa, respectively. However, there was no evidence in the gel for their overexpression using the *P*_*cpt*_ promoter. This suggested low accumulation of the heterologous enzymes under the control of *Pcpt* promoter [2, 26]. Nevertheless, the strong redirection of the carotenoid biosynthetic pathway toward astaxanthin in the BC strain (Fig. 3g) confirms that the enzymatic activities of bKT and CrtZ were sufficient to achieve the desired metabolic engineering, with no need to further increase the accumulation of bKT and CtrZ enzymes to boost ketocarotenoid accumulation. To prove the stable expression of the BC DNA operon under the control of the *P*_cpt_ promoter, RT-PCR analyses were conducted using the cDNA sequences derived from the BC cultures grown for different days in airlift PBRs as templates. In more detail, algal cultures were grown for 3, 4, 7 and 10 days under high-light condition (see also Fig. 5a). The endogenous constitutive *rpnA* gene transcript was expressed in each sample, as expected. Similarly, BC operon expression, evaluated through the amplification of *crtZ* gene transcript, was observed in each tested condition (Supplementary Figure S3c, Additional File 1). The primers used for the amplification of the *crtZ* gene transcript are listed in Table 1.

**Fig. 4:**
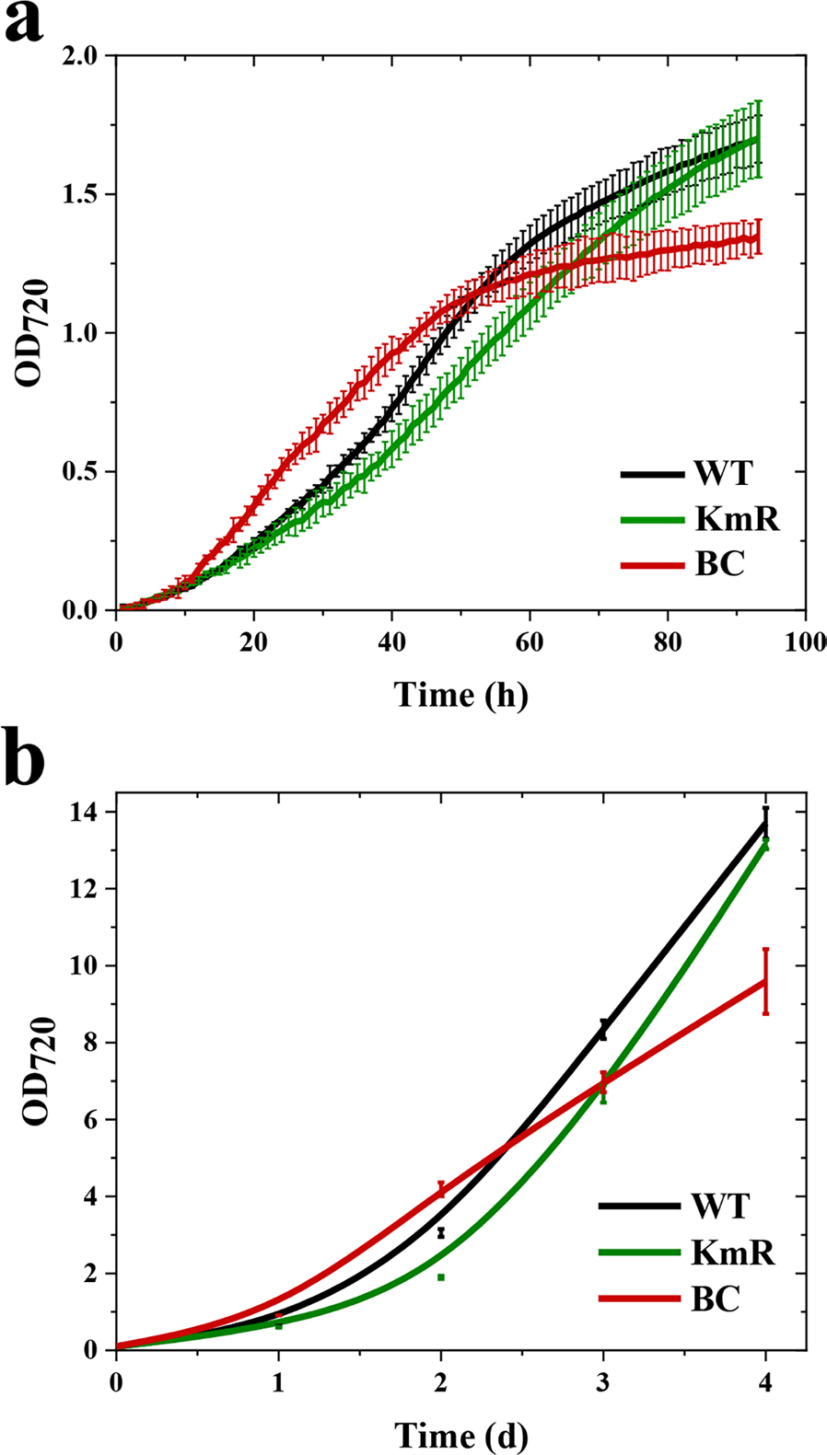
Effect of the accumulation of non-native astaxanthin on the microalgal growth. **a** Growth curves of WT, KmR and BC lines cultivated in batch in the 80-ml airlift PBRs, using OD_720_ as an index of cell density. Cultures were exposed to 300 µmol/m^2^/s photons of white light for the first 8 h, then light was increased from 300 to 1500 µmol/m^2^/s in 12 h. Eventually, light was kept at 1500 µmol/m^2^/s until the end of the experiment. WT and KmR lines served as controls. Two independent cultures per condition were evaluated. Error bars represented standard deviation (*n* = 4). **b** Manual OD_720_ measurements, conducted on a daily basis, using diluted samples to avoid saturation. This analysis was in agreement with automatic OD-monitoring of the device used for cyanobacterial cultivations (a). Two independent cultures per condition were evaluated. Error bars represented standard deviation (*n* = 4)

To evaluate the impact of Asta accumulation on *Syn11901* growth rate, WT, KmR and BC strains were cultivated in the airlift PBRs using the same conditions previously adopted for bKT lines using 1500 μmol/m^2^/s of light and 3% (v/v) CO_2_ (Supplementary Figure S4a, Additional File 1). BC lines were characterized by a faster growth rate than WT and KmR lines (Fig. 4a, b), with stationary phase reached after ~ 50 h of growth compared to more than 80 h for WT and KmR. KmR cultures showed slightly delayed growth compared to WT, in agreement with previous literature showing that the replacement of the *acsA* locus causes a reduction in growth rate [27], although in stationary phase WT and KmR cultures reached similar ODs.

Table 2 reports the dry biomass accumulated by the different cultures at days 4 and 7: the highest biomass accumulation was obtained for the WT and KmR lines, which reached ~ 3.4 g/L at day 4 and ~ 7.8 g/L. BC cultures accumulated less biomass at day 7 than the other two strains. Absorption spectra of acetone pigment extracts obtained from BC cells harvested on day 4 or 7 indicated enhanced absorption at ~ 500 nm attributable to ketocarotenoids (Supplementary Figure S4b, Additional File 1). HPLC analysis confirmed the Asta accumulated to ~ 0.4% of total cell biomass in BC cultures on both day 4 and 7 (Supplementary Figure S4c, Additional File 1). As a further confirmation of the synthesis of Asta in the BC strain, a preparative thin-layer chromatography (TLC) was performed. The TLC profile in the BC strain was in agreement with the HPLC analysis, considering the abundant red band present only in the BC extract which possibly represented Asta (Supplementary Figure S4d, Additional File 1). The eluted pigment in acetone 80% had a maximum of absorption at 480 nm, same as previously described for astaxanthin (Supplementary Figure S4e, Additional File 1). In addition, the eluted fraction was analyzed by mass spectrometry. Interestingly, two peaks referring to Asta were identified, the protonated molecule [M + H]^+^ at *m/z* 597 and the metal adduct ion [M + Na]^+^ at *m/z* 619 (Supplementary Figure S4f, Additional File 1), as described in literature [36].

**Table 2:** Biomass and Asta accumulation in WT, KmR and BC cultures grown in the airlift PBRs

Strain	Growth (days)	Asta (mg/L)	dcw (g/L)	Asta/dcw (%)	Asta/car (%)	Chl*a*/dcw (%)
WT	4	nd	3.47 ± 0.11	nd	nd	1.91 ± 0.10
	7	nd	7.80^a^ ± 0.19	nd	nd	1.76^a^ ± 0.07
KmR	4	nd	3.39 ± 0.18	nd	nd	1.94 ± 0.13
	7	nd	7.83^a^ ± 0.36	nd	nd	1.72^a^ ± 0.13
BC	4	14 ± 4	3.17 ± 0.14	0.44^a^ ± 0.08	84.1^a^ ± 2.5	0.56 ± 0.14
	7	29 ± 4	6.59^b^ ± 0.15	0.44^a^ ± 0.04	86.7^a^ ± 3.1	0.61^b^ ± 0.10

*nd* not detected, *dcw* dry cell weight, *car* total carotenoids, *Chla* chlorophyll *a*; ± represents standard deviation. Statistical significance is expressed by different letters according to Tukey–Kramer test (*n* = 8)

Differently from Asta, Chl*a* content was decreased in the BC line compared to both WT and KmR strains (Table 2), which is consistent with the phenotype of *C. reinhardtii* strains engineered to accumulate Asta [19]. This evidence was corroborated by whole-cell absorption spectra taken and normalized to the chlorophyll content (Supplementary Figure S4g, Additional File 1). WT and KmR spectra were, as expected, similar, whereas BC samples showed enhanced absorption, especially in the blue region of visible light. This is attributable to greater light scattering, a proxy of a greater cell density, in BC cultures compared to those WT and KmR, containing the same chlorophyll content.

**Fig. 5:**
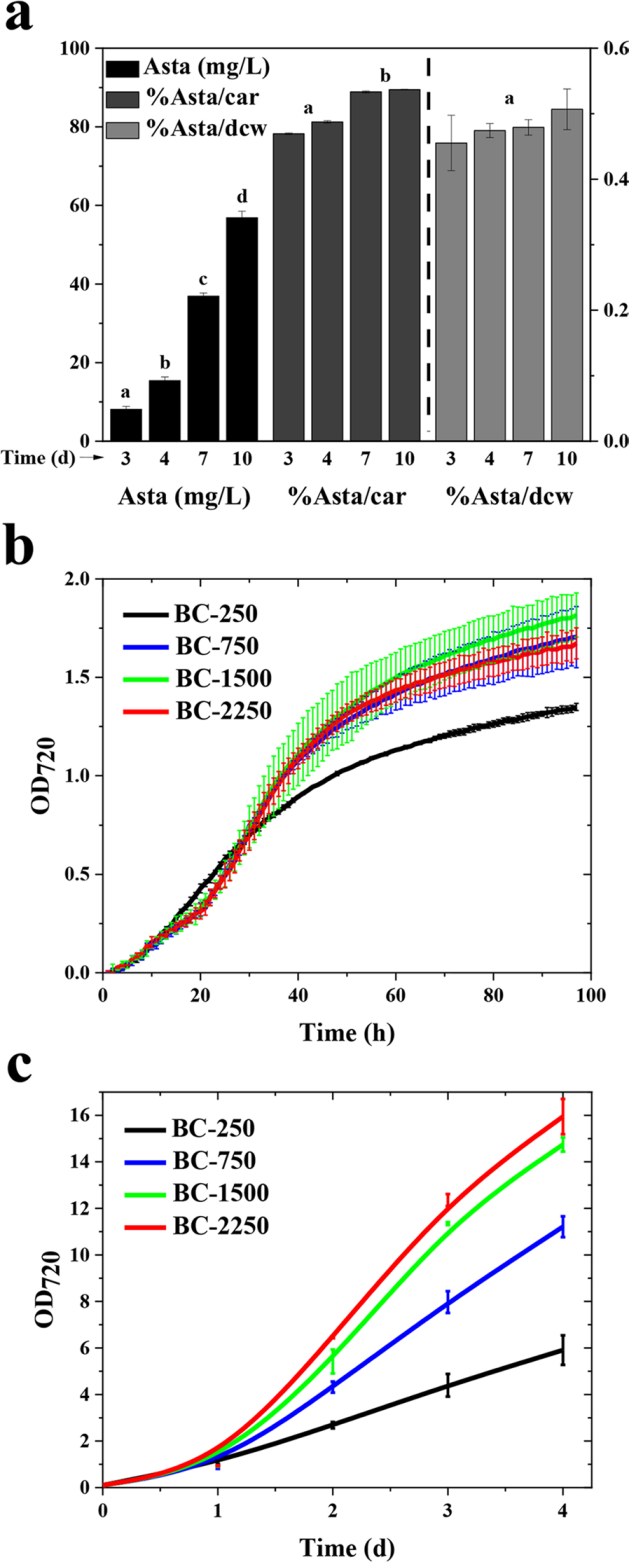
Astaxanthin and biomass accumulation of cyanobacterial transformant. **a** Accumulation of astaxanthin in BC cultures grown in batch mode for 10 days in 80-ml airlift PBRs. Samples were collected on days 3, 4, 7, and 10. Cells were exposed to an initial gradient of white light, reaching 1500 µmol/m^2^/s at day 2. Statistical significance is expressed by different letters according to Tukey–Kramer test (*n* = 4). **b** Growth curves of BC lines cultivated in batch mode in 80-ml airlift PBRs, using OD_720_ as an index of cell density. Cultures were exposed to 250 µmol/m^2^/s of white light for the first 6 h. The light was then increased following a stepwise gradient (1 h to increase light intensity to the next level of light intensity, which was kept for 6 h, and then this whole step was further replicated until reaching the desired light intensity). Final light intensities reached in the 4 tested conditions were 250 µmol/m^2^/s, 750 µmol/m^2^/s, 1500 µmol/m^2^/s, and 2250 µmol/m^2^/s. Two independent cultures per condition were evaluated. Error bars represented standard deviation (*n* = 4). **c** OD_720_ of samples described in **a**: samples were diluted accordingly below 1 before measurements to avoid saturation. Error bars represented standard deviation (*n* = 4)

Accumulation of Asta was monitored in a batch culture of BC cells grown in airlift PBRs with a final light intensity of 1500 μmol/m^2^/s, and cells were sampled at days 3, 4, 7 and 10 of growth. The Asta content (mg/L) increased over-time (Fig. 5a) and % Asta/car was higher than 80% in each condition. Most importantly, % Asta/dcw did not substantially vary in the evaluated phases of the cyanobacterial cultivation.

To better understand the relationship between the accumulation of biomass and Asta, a BC culture was grown under stepwise light gradients in airlift PBRs, reaching final light intensities of 250, 750, 1500, or 2250 µmol photons/m^2^/s (Fig. 5b). These experiments revealed a correlation between light intensity, cell growth (Fig. 5b, c) and biomass accumulation (Table 3): 4 days growth at 250 µmol/m^2^/s resulted in ~ 2.2 g/L of dry cyanobacterial biomass (dry cell weight, dcw), whereas the 2250 µmol/m^2^/s culture generated ~ 7.5 g dcw/L. Accordingly, the biomass surface productivity, calculated based on the surface area of PBRs exposed to light, increased from 9 to 31.3 g/m^2^/day (Table 3) upon increasing the light intensity. The areal productivity recorded was higher compared to other microalgal records reported in the literature [37], even considering optimized condition in a semicontinuous cultivation system [38]. Potentially, higher biomass productivity could be obtained upon continuous cultivation [39].

**Table 3:** Relation among light intensity, surface biomass productivity, biomass and Asta accumulation in BC cultures grown in the airlift PBRs

	Light intensity (µmol/m^2^/s)	Biomass (g/L)	Surface productivity (g/m^2^/day)	Asta (mg/L)	Asta/dcw (%)	Asta/car (%)	Chl*a*/dcw (%)
80 mL PBRs	250	2.2 ± 0.2	9.0 ± 0.4	11.5^c^ ± 1.1	0.52^a^ ± 0.04	83.9^a^ ± 0.7	1.5^a^ ± 0.1
	750	4.4 ± 0.1	18.1 ± 0.4	24.7^b^ ± 0.7	0.56^a^ ± 0.01	86.3^a,b^ ± 1.4	1.7^a^ ± 0.1
	1500	6.4 ± 0.2	26.3 ± 0.5	37.0^a^ ± 3.6	0.58^a^ ± 0.05	88.4^b^ ± 1.7	1.2^b^ ± 0.1
	2250	7.5 ± 0.6	31.3 ± 0.9	38.4^a^ ± 3.4	0.52^a^ ± 0.04	89.7^b^ ± 3.1	0.9^b^ ± 0.2
330-L PBR	800	1.0 ± 0.2	16.7 ± 1.7	4.1 ± 1.2	0.4 ± 0.07	73.3 ± 1.9	2.3 ± 0.2

*Car* total carotenoids, *Chla* chlorophyll *a*, *dcw* dry cell weight; ± represents standard deviation. Statistical significance is expressed by different letters according to the Tukey–Kramer test (*n* = 8 except for the data obtained in 330L-PBR where *n* = 4)

The absorption spectra of acetonic extracts from samples collected after 4 days of cultivation were taken. Such spectra, after normalization to the Chl*a* content, showed an absorption at 480 nm, attributable to carotenoids, that correlated with light intensity (Supplementary Figure S5, Additional File 1). Conversely, spectra normalized to the carotenoid content evidenced a very similar absorption attributable to carotenoids (Supplementary Figure S5, Additional File 1). This suggested a similar carotenoid composition of carotenoids accumulating in the photosynthetic membranes of BC cells grown under the different light conditions.

Astaxanthin accumulation on a volume basis was greater at high light intensities, reaching ~ 38.4 mg/L in 2250 µmol/m^2^/s samples after 4 days of growth. Moreover, Asta represented more than 80% of total carotenoid (Table 3). The Chl*a*/biomass ratio content was lower in the culture grown at higher light intensities, suggesting a reduction of photosystems per cell in response to the greater photon availability. The increased growths at higher light intensities, in the presence of a non-limiting amount of CO_2_, showed that *Syn11901* could tolerate very high light with a surface productivity that, under the conditions tested, was close to 30 g/m^2^/day. From the productivity analysis (Table 3), it emerged that photosynthetic active cells of *Syn11901* produce up to ~ 0.6% of astaxanthin on cell dry weight against the 3 mg/g (0.3%) reported for the closely related *Synechococcus* PCC 7002 [15].

### Astaxanthin accumulation is not triggered by nutrient starvation

Nutrient starvation is known to boost astaxanthin accumulation in several algal species, such as *H. lacustris* [10] or *C. zofingiensis* [12].

**Fig. 6:**
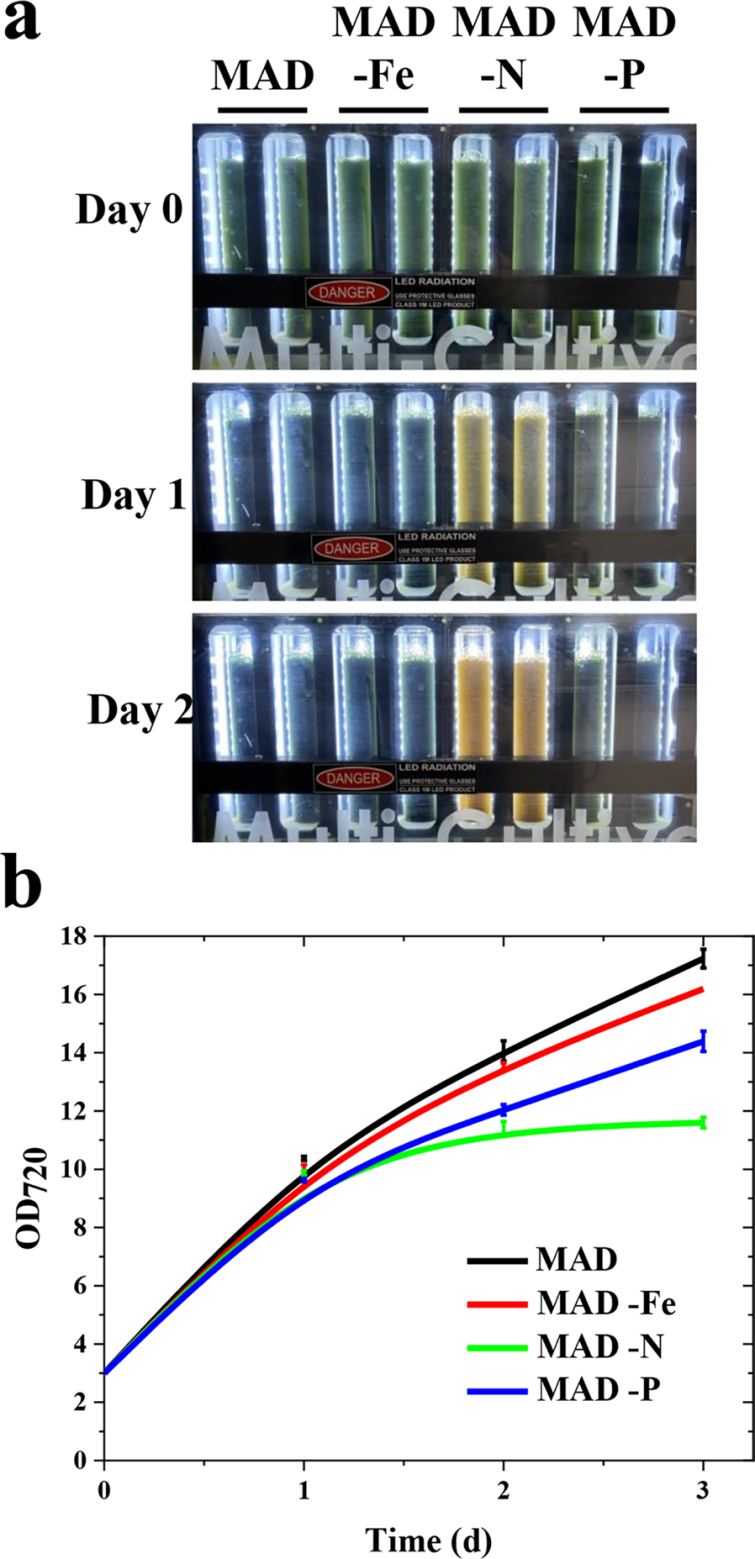
Nutrient starvation of BC cultures in airlift PBRs. **a** Growth of BC cultures cultivated in batch mode in 80-ml airlift PBRs in the absence of specific nutrients, such as iron (Fe), nitrogen (N) and phosphorus (P). BC cells were previously grown in MAD medium reaching stationary phase, and then cells were washed and resuspended to OD_720_ of 3 in the different media. MAD medium was used as a control. Pictures were taken on days 0, 1, and 2. **b** OD_720_ of samples described in **A**: samples were diluted accordingly before measurements to avoid saturation. Error bars represented standard deviation (*n* = 4)

To evaluate the effect of nutrient starvation on astaxanthin productivity in the engineered *Syn11901*, the BC strain was cultivated in replete MAD medium or MAD medium deprived of a specific nutrient inducing nitrogen, phosphorus or iron starvation (see Materials and methods section). After 3 days of growth at 2250 µmol photons/m^2^/s, nitrogen starvation caused a decrease in biomass productivity compared to the nutrient-replete condition (Fig. 6a, b). However, the Asta concentration per dry weight was similar or even lower compared to the control condition (Table 4). The negative effect on biomass accumulation was less evident in cultures depleted of phosphorus or iron, suggesting a sufficient endogenous pool of the two elements.

**Table 4:** Evaluation of the effect of nutrient starvation on Asta accumulation in BC cultures grown in the airlift PBRs shown in Fig. 6

Medium	Growth (days)	Asta (mg/L)	dcw (g/L)	Asta/dcw (%)	Chl*a*/dcw (%)
MAD	3	36.71^a^ ± 0.31	7.49^a^ ± 0.02	0.49^a,b^ ± 0.06	0.76^a^ ± 0.03
MAD-N	2	19.35^b^ ± 0.51	3.95^c^ ± 0.14	0.49^a,b,c^ ± 0.10	0.42^b^ ± 0.08
	3	15.24^c^ ± 0.74	4.62^d^ ± 0.34	0.33^c^ ± 0.06	0.27^c^ ± 0.05
MAD-Fe	3	35.04^a^ ± 0.41	6.74^b^ ± 0.12	0.52^b^ ± 0.03	0.80^a^ ± 0.04
MAD-P	3	28.82^d^ ± 0.32	6.55^b^ ± 0.24	0.44^a^ ± 0.04	0.65^d^ ± 0.04

Chl*a*, chlorophyll *a*, *dcw* dry cell weight; ± represents standard deviation. Statistical significance is expressed by different letters according to the Tukey-Kramer test (*n* = 4)

Exposure to nutritional stressing conditions is thus not an advantageous strategy to boost Asta productivity in *Syn11901* BC strain. Differently, in the case of endogenous Asta accumulation observed in *H. lacustris* [10] or *C. zofingiensis* [12], Asta accumulation is specifically triggered to overcome oxidative stress in conditions where the photosynthetic activity is strongly downregulated. Optimization of Asta production by *Syn11901* BC strain is thus related to improving carbon fixation and biomass productivity and continuing the metabolic engineering of *Syn11901*.

### Up-scaling of cyanobacterial cultivation

**Fig. 7:**
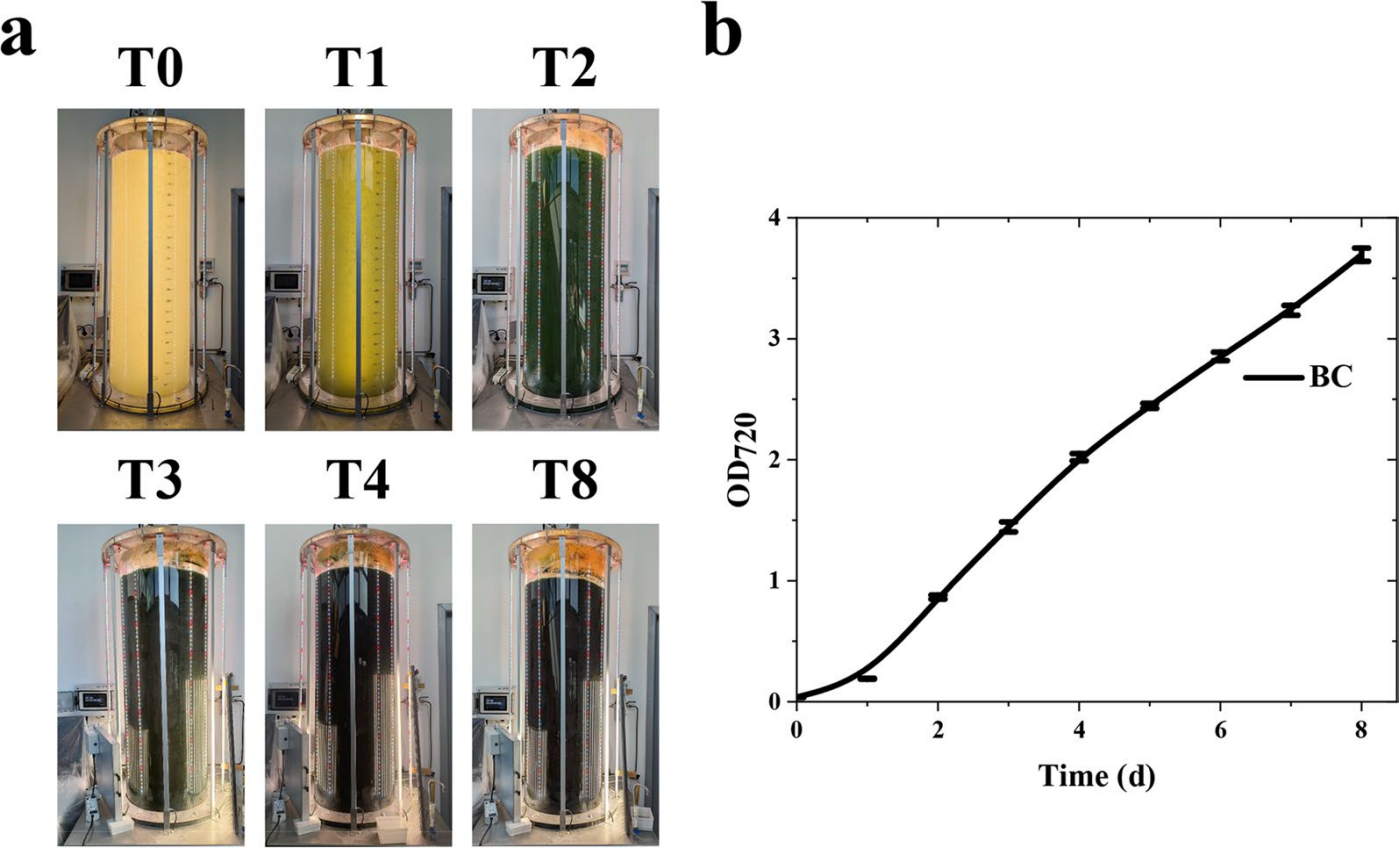
Cultivation of BC cells in an industrial PBR. **a** BC strain was photoautotrophically grown in batch mode in a 330L-PBR with limited sterility. The numbers on top of each picture indicate the day of growth. **b** Biomass accumulation curve of BC strain, as measured from the optical density of the cultures at 720 nm. Cultures were inoculated to an initial cell concentration corresponding to OD_720_ 0.037 ± 0.003. Error bars represent standard deviation (*n* = 4)

Next, cultivation of the BC strain was scaled up to a 330-L PBR. The PBR was air-bubbled for cell mixing and supplied with pure CO_2_ on demand to maintain the desired pH (see Material and methods). Cells were inoculated to an OD_720_ of 0.033, a lower value compared to the previous experiments conducted in the 80-ml PBRs. Despite the low cyanobacterial inoculum, the color of the culture already shifted from a pale yellow to a more vivid green after 1 day of cultivation, indicating photosynthetic activity (Fig. 7a). After 4 days of cultivation, the culture had a dark coloration, suggesting a continuous growth of the BC strain. No clear visible differences in pigmentation of the culture were present from 4 to 8 days of growth, although cell density continuously increased during this time span, as evidenced by OD_720_ measurements (Fig. 7b). However, the measured OD_720_ was lower compared to what observed in the 80-ml PBRs (Fig. 5c). In the latter, OD_720_ ranged between 5 and 15 after 4 days of growth, depending on the light irradiance, whereas, in the case of the 330-L PBR, the increase of biomass accumulation slightly slowed down after 4 days of growth, reaching an OD_720_ of ~ 3.8 after 8 days of cultivation (Fig. 7b).

After 8 days of growth, the cyanobacterial biomass was ~ 1 g dcw/L with a biomass surface productivity of 16.7 g/m^2^/day and a final Asta accumulation of 0.4% of total dry biomass (Table 3). Despite the lower biomass concentration obtained compared to the 80-mL PBR growth reported above, the surface productivity was in the range of the surface productivity obtained upon illumination with 750 µmol/m^2^/s, indicating that light penetration in the large-scale 330-L PBR was the limiting factor for biomass accumulation.

## Discussion

The advancements in synthetic biology promoted the generation of alternative microalgal strains [15–21, 40, 41], which were genetically modified with the aims of synthesizing non-native Asta and, most importantly, overcoming limitations of *H. lacustris*. Cyanobacterial platforms had the advantage over eukaryotic microalgae of efficiently synthesizing a smaller pool of carotenoids, mainly βcar and Zea, the precursors of microalgal Asta [22].

The first step of the experimental effort described in this manuscript was inserting of the *bKT* construct (Fig. 2a) in the *acsA* locus, whose sequences for HR were already available [2]. The obtained transformant was characterized by a brownish pigmentation (Fig. 3a), similarly to other engineered microalgae accumulating ketocarotenoids [15–21, 40–42]: accordingly, HPLC analysis demonstrated the main accumulation of Cantha in the bKT transformant, while Asta was found only as a minor fraction of total carotenoids. This finding demonstrates that the activity of the endogenous hydroxylation activity of CrtZ enzyme was limiting the conversion of Cantha to Asta. Thus, a second round of transformation was conducted, replacing the kanamycin-resistance cassette of bKT transformant with *crtZ* from *Brevundimonas* sp. SD-212 [17] and *smR* genes, with the latter conferring resistance to spectinomycin, in an operon configuration. The choice for the use of *crtZ* from *Brevundimonas* sp. SD-212 over other possible βcar hydroxylases was due to previous literature about the efficiency of this enzyme in converting Cantha to Asta [17, 33]. Moreover, the prokaryotic origin of the *crtZ* gene herein adopted mitigate the risk of inefficient heterologous gene expression observed in some cases expressing eukaryotic genes in cyanobacteria [43, 44]. HPLC analysis confirmed that Cantha was almost absent in BC extract, being entirely replaced by Asta (Fig. 3g). Remarkable was that the SDS-PAGE analysis of total protein extracts from the evaluated lines (Supplementary Figure S3, Additional File 1) showed no bands attributable to the recombinant bKT and CrtZ enzymes. This suggested low accumulation of the heterologous enzymes under the control of *Pcpt* promoter [2, 26]. Nevertheless, transcripts analysis demonstrated that the enzymes were expressed (Supplementary Figure S3c, Additional File 1) successfully redirecting carotenoid biosynthesis. Anyway, other genetic tools could be evaluated in *Syn11901* to further boost recombinant enzymes expression because the success of synthetic biology approaches generally requires true overexpression of pathway enzymes and proteins of interest to attain higher yields and lower costs [44].

Importantly, the BC line displayed the fastest growth in exponential phase, despite replacement of *acsA* (Fig. 4). Thus, the presence of astaxanthin did not negatively impact growth of the BC line despite the strong reduction of Zea and βcar in the *Syn11901* BC strain. Zea is usually found in lipid membranes having a role in photoprotection in cyanobacteria [45], but this role is likely complemented by Asta in *Syn11901* BC strain. βcar is essential for photosystems assembly, but the residual βcar is likely sufficient for ensuring the accumulation of the photosynthetic complex required for photoautotrophic growth of *Syn11901 BC* strain. Rather, *Syn11901 BC* was characterized by a ‘boost’ in the initial stages of growth, with a faster growth rate in the exponential phase compared to WT. This effect is consistent with previous growth data for *C. reinhardtii* engineered to accumulate Asta [46] and could be due to: (1) the antioxidant properties of astaxanthin protecting cells and photosystems in the early exponential phase, where the culture is relatively dilute [46, 47] and/or (2) the reduced amount of chlorophyll per cell (Table 2), conferring a pale-green-like phenotype, which favors greater light penetration, thereby enhancing photosynthetic efficiency [34].

The yield of Asta produced under the best conditions tested resulted in 38.4 mg/L after 4 days of cultivation (Table 3) at an average rate of production of ~ 9.6 mg/L/day, which exceeds that for *H. pluvialis*, with reported yields in the range of 0.12–4.4 mg/L/day using tubular or bubble columns [48]. Even if a higher production yield could be obtained for *H. pluvialis* in more complex cultivation systems [49, 50], increasing light availability could further increase the Asta production yield even in the case of the BC strain herein reported. Asta production in *Syn11901* is also substantially higher than Asta heterologously synthesized in the cyanobacterium *Synechocystis* sp. PCC 6803 (2.8 mg/L/day [21]) and the green alga *C. reinhardtii* (6.96 mg/L/day; [32]). Engineered yeasts produce Asta at rates of 37.5 mg/L/day [51], but growth is heterotrophic and relies on adding glucose to the media. A summary of the production yield obtained in this work compared to other systems is reported in Table 5. One of the advantage in using *Syn11901* compared to other systems is the fact that most carotenoids are present in this species as zeaxanthin and βcar. These molecules are the substrates of the bKT and CtrZ enzymes introduced, allowing for efficient production of Asta (Fig. 1). Other carotenoids are strongly accumulated in other systems, such as *Chlamydomonas reinhardtii* or *Nannochloropsis*, providing competitive sinks for the precursors needed for astaxanthin biosynthesis. As reported in Table 3, the lowest Asta content per dry weight was observed in cells grown at the lowest irradiances in 80-ml photobioreactors or in cells grown on a 330-L scale, where light penetration is strongly limited (Table 3): exposure to sufficient light is thus needed to boost astaxanthin content per dry weight. Even if nutritional stress did not provide any improvement in Asta titer (Table 4), we cannot exclude that other stressing conditions might somehow improve carotenoids biosynthesis.

**Table 5:** Summary of the astaxanthin productivity and content per dry weight in different production systems

Genotype	Productivity	Asta/dcw	References
	(mg/L/day or mg/m^2^/day)	%	
*Synechococcus* sp. PCC 11901	9.6 mg/L/day; 165.4 mg/m^2^/day	0.59	This work
*Haematococcus pluvialis*	up to 4.4 mg/L/day;	up to 1.8	[48]
*Haematococcus pluvialis*	204 mg/m^2^/day	3.8	[50]
*Haematococcus pluvialis*	15.84 mg/L/day	4.9	[49]
*Synechocystis* sp. PCC 6803	2.8 mg/L/day	0.30	[21]
*Chlamydomonas reinhardtii*	6.96 mg/L/day	0.45	[32]
*Nannochloropsis oceanica*	9.9 mg/L/day in fed-batch mode	0.73	[41]
*Saccharomyces cerevisiae*	37.5 mg/L/day	1.4	[51]
*Yarrowia lipolytica*	275 mg/L/day in fed-batch mode	4.1	[56]

There is also scope to improve the production of Asta in the BC strain through additional metabolic engineering and improved PBR design. For instance, levels of isopentenyl pyrophosphate (IPP) and dimethylallyl pyrophosphate (DMAPP) which are the precursors of carotenoids and other terpenoids could be enhanced [21, 32, 44, 52–54]. Given that deletion of *acsA* might have a potential negative impact on the growth of the BC strain new *loci* for insertion of the *bKT* and *crtZ* genes could also be tested [4]. Regarding the cultivation of the engineered strain, the scaling-up costs can be reduced thanks to the recent isolation of a cobalamin-independent strain of *Syn11901* that does not require the addition of vitamin B12 [3].

Possible cultivation in outdoor systems using natural sunlight could also be considered as a possible strategy to further reduce the production costs, even if the cultivation of GMO strains, such as *Syn11901 BC* is subject to strict regulation.

The high salinity of the MAD medium also represents a barrier for contamination by bacteria, weedy algae/cyanobacteria and other organisms, during the cultivation process. However, this barrier can be further strengthened by introducing the PtxD/phosphite-utilizing system [55] in *Syn11901*, which was shown to allow cyanobacterial productions in non-sterile outdoor reactors, reducing costs. In terms of PBR design, improving light penetration, for example by using a tubular PBR, will improve photosynthetic performances and, consequently, biomass and Asta yields.

## Conclusions

The metabolic engineering approach herein reported in *Syn11901* lead to Asta production at rates of ~ 10 mg/L/day under photoautotrophic growth conditions without the need for stress conditions such as nutrient starvation. Moreover, *Syn11901* produces phycocyanin, another industrial-relevant product with different applications in the food and cosmetics sectors. Thus, a biorefinery process to produce ketocarotenoids and phycocyanin in the *Syn11901* BC strain is a promising industrial strategy. Furthermore, considering the high photosynthetic efficiency of this fast-growing strain, its cultivation could be integrated with CO_2_-emitting processes for carbon sequestration and conversion into high-value products such as Asta.

## Supplementary Information

Supplementary material 1: Figure S1. Absorption spectra in the visible light range of eluted fractions from HPLC analysis of extracts from WT and bKT cultures, as shown in Figure 2f. Figure S2. Comparison of astaxanthin accumulation in bKT transformants grown in flasks or in 80 ml airlift photobioreactors. Figure S3. Protein expression and RT-PCR analysis of *Syn11901 *wild type and transformants. Figure S4. HPLC, TLC and MS analysis of BC strain. Figure S5. Absorption spectra in the visible light range of pigments extracts from cultures grown 4 days in airlift PBRs and described in Figure 6.

## Data Availability

All data generated or analyzed during this study are included in this published article and its supplementary information files. The DNA sequences used for metabolic engineering can be found at this link: 10.5281/zenodo.13234572. MS results can be found at this link: 10.5281/zenodo.14283202.
