# Supplementary material for: Engineering of the fast-growing cyanobacterium Synechococcus sp. PCC 11901 to synthesize astaxanthin
Source: Biotechnol Biofuels Bioprod. 2025 Feb 28;18:28. doi: 10.1186/s13068-025-02626-5 (PMC11871721; doi:10.1186/s13068-025-02626-5)
Supplement: Supplementary file 1 — Supplementary material 1: Figure S1. Absorption spectra in the visible light range of eluted fractions from HPLC analysis of extracts from WT and bKT cultures, as shown in Figure 2f. Figure S2. Comparison of astaxanthin accumulation in bKT transformants grown in flasks or in 80 ml airlift photobioreactors. Figure S3. Protein expression and RT-PCR analysis of Syn11901 wild type and transformants. Figure S4. HPLC, TLC and MS analysis of BC strain. Figure S5. Absorption spectra in the visible light range of pigments extracts from cultures grown 4 days in airlift PBRs and described in Figure 6. [file 13068_2025_2626_MOESM1_ESM.pdf]

Supplementary Materials

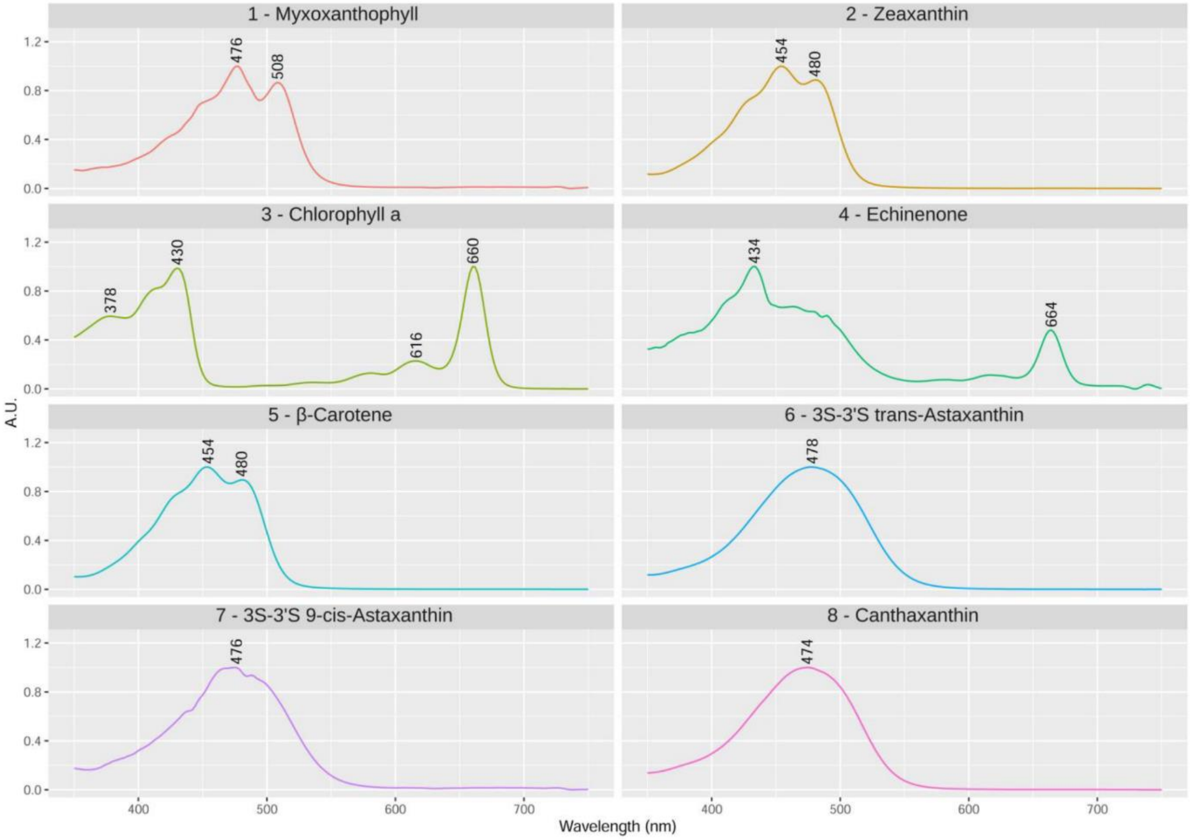

Supplementary Figure 1. Absorption spectra in the visible light range (350-750 nm) of eluted fractions from HPLC analysis of extracts from WT and bKT cultures, as shown in Figure 2f. 1, mixoxanthophyll; 2, zeaxanthin; 3, chlorophyll *a*; 4, echinenone (fraction contaminated with chlorophyll *a*); 5, β-carotene; 6, 3S,3'S trans-Astaxanthin; 7, 3S,3'S 9-cis-Astaxanthin; 8, canthaxanthin.

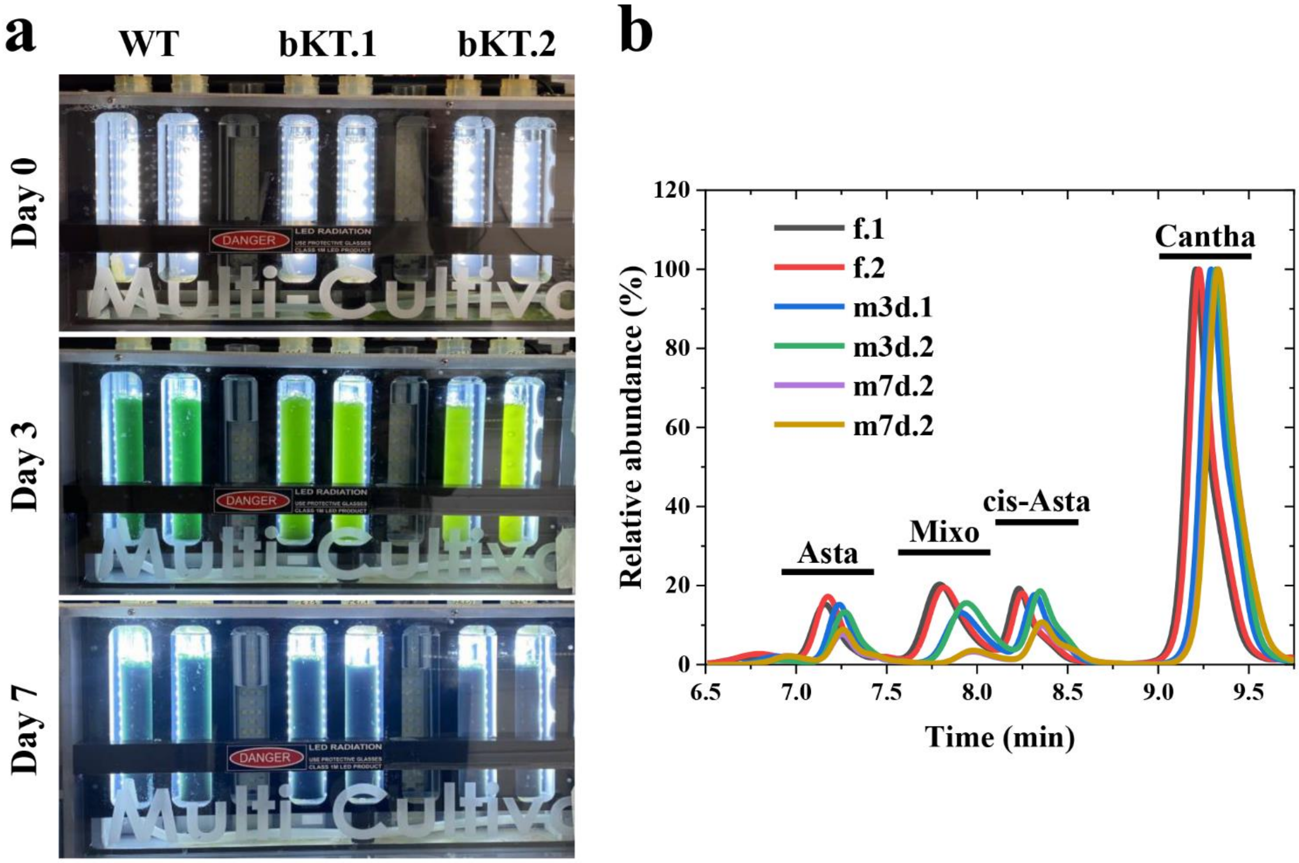

Supplementary Figure 2. Comparison of astaxanthin accumulation in bKT transformants grown in flasks or in 80 ml air-lift photobioreactors. **(a)** Growth of WT and bKT lines in air-lift PBRs in a Multicultivator system exposed to 1500 µmol/m^2/s irradiance with 3% CO_2-enriched air supply. Pictures were taken on days 0, 3, and 7. **(b)** Relative abundance of ketocarotenoids in bKT transformants grown in flasks, as shown in Figure 2d, and upon 7 days of growth in a Multicultivator system. HPLC chromatograms were normalized to the peak attributed to canthaxanthin. Asta, 3S,3'S trans-Astaxanthin; Mixo, mixoxanthophyll; cis-Asta, 3S,3'S 9-cis-Astaxanthin; Cantha, canthaxanthin.

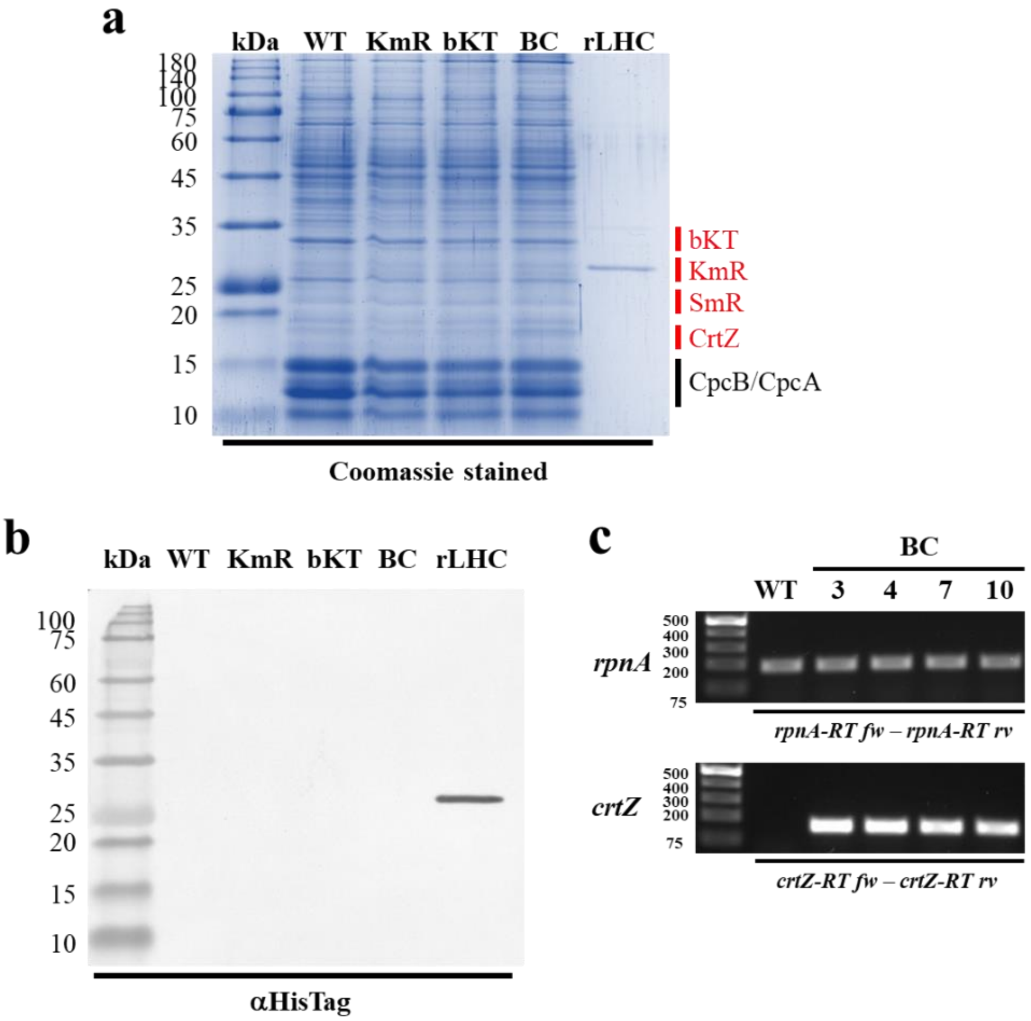

Supplementary Figure 3. Protein expression and RT-PCR analysis of *Syn11901* wild type and transformants. **(a)** Total cellular protein extracts from WT, KmR, bKT, and BC cells were resolved by SDS-PAGE and visualized by Coomassie staining. Sample loading corresponds to 0.45 µg of chlorophyll. CpcB and CpcA subunits of phycocyanin were recognized as the most abundant polypeptides and marked with a black bar, whereas putative migration regions of heterologous proteins were marked in red. A recombinant his-tagged protein (rLHC) was used as a control. (**b**) Total protein extracts of (a) were subjected to Western-blot analysis with loading of the lanes as above. Specific polyclonal antibodies against the his-tag domains were used to probe target proteins. (**c**) RT-PCR analysis for verifying the expression of transgenic *crtZ* gene and constitutive *rpnA* gene transcripts in WT and BC cell cultures. The PCR products were separated on 1.5% agarose gel. The expected size of the PCR products was 152 and 231 bp for *crtZ* and *rpnA* gene transcripts, respectively. Numbers represent the days of growth.

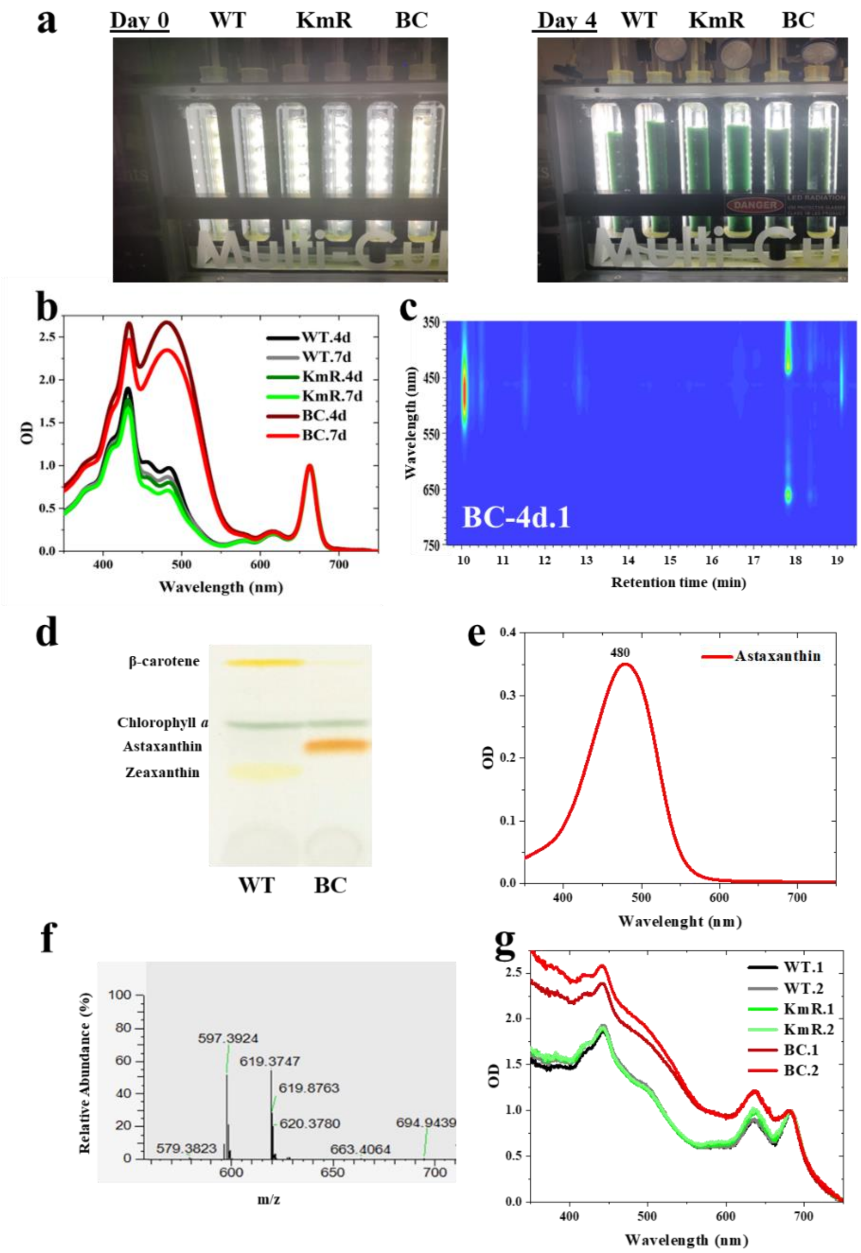

Supplemental Figure 4. HPLC, TLC and MS analysis of BC strain. **(a)** Cultivation of WT, KmR and BC cells in air-lift PBRs. Pictures were taken at day 0 and 4. **(b)** Representative absorption spectra (350-750 nm) of DMSO-acetone extracts from WT, KmR, and BC cells grown in air-lift PBRs. Cells were harvested after 4 (4d) and 7 days of growth (7d). **(c)** HPLC analysis of a representative sample of BC extracts obtained from cells grown for 4 days in an air-lift PBR. **(d)** Thin Layer Chromatography of isopropanol extracts from wild-type (WT) and BC cells. **(e)** Absorption spectrum of Asta fraction eluted from (d) in acetone 80%. Maximum of absorption was at 480 nm. **(f)** Mass Spectrometer analysis of eluted Asta fraction from (d). Identified ions derived from Asta were the protonated molecule $[M+H]^+$ at m/z 597 and the metal adduct ion $[M+Na]^+$ at m/z 619. **(g)** Whole-cell spectra in the visible range of light (350-750 nm) of cultures grown in air-lift PBRs.

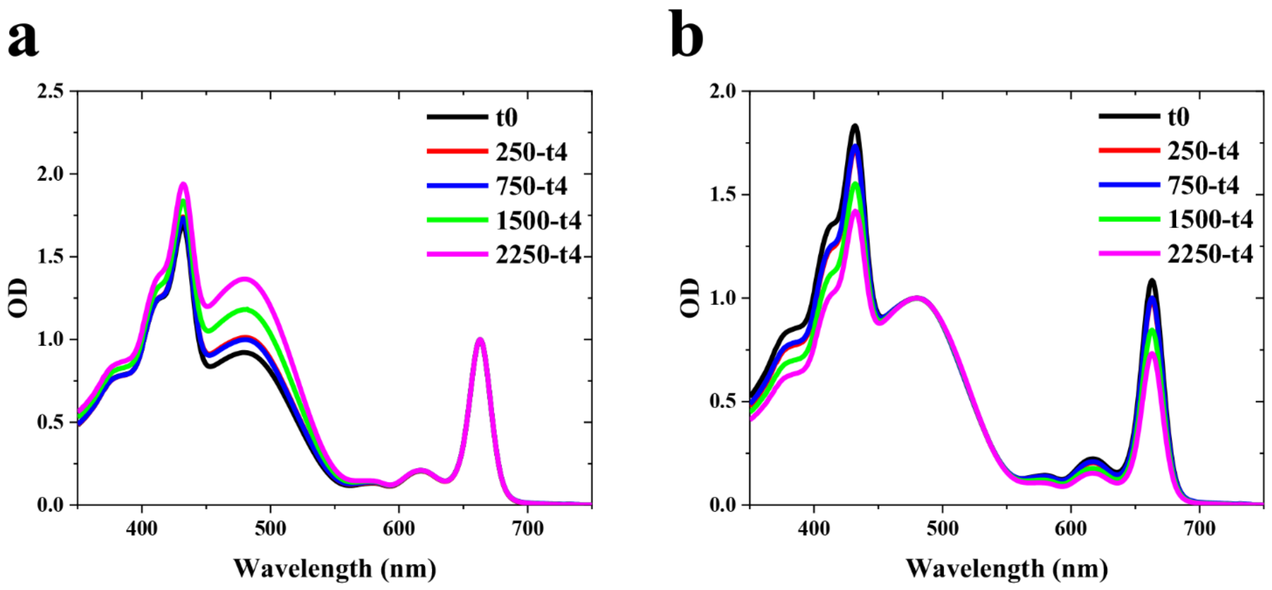

Supplementary Figure 5. Absorption spectra in the visible range of light (350-750 nm) of pigments extracts from cultures grown 4 days in air-lift PBRs and described in Figure 6. The number comprised in each name of the different samples indicates the light intensity used for the cultivation (μmol/m^2/s), whereas “t” represents the day of cultivation. **(a)** Spectra normalized to the Chl*a* content. **(b)** Spectra normalized to the carotenoid content.
